# Serotonergic Innervation of the Salivary Glands and Central Nervous System of Adult *Glossina pallidipes* Austen (Diptera: Glossinidae), and the Impact of the Salivary Gland Hypertrophy Virus (GpSGHV) on the Host

**DOI:** 10.1093/jisesa/iev162

**Published:** 2016-01-21

**Authors:** Laura Guerra, John G. Stoffolano, Maria Cristina Belardinelli, Anna Maria Fausto

**Affiliations:** ^1^Dipartimento per la Innovazioni nei sistemi Biologici, Agroalimentari e Forestali, Università degli Studi della Tuscia, Largo dell’Università snc, 01100 Viterbo, Italy (lauraguerra@unitus.it; belardinelli@unitus.it; fausto@unitus.it),; ^3^Stockbridge School of Agriculture, 270 Stockbridge Rd., Fernald Hall, Room 204A, University of Massachusetts, Amherst, MA 01003, USA (stoff@ent.umass.edu)

**Keywords:** serotonin, *Glossina pallidipes*, GpSGHV, neuronal proliferation, salivary gland muscle sheath

## Abstract

Using a serotonin antibody and confocal microscopy, this study reports for the first time direct serotonergic innervation of the muscle sheath covering the secretory region of the salivary glands of adult tsetse fly, *Glossina pallidipes* Austen. Reports to date, however, note that up until this finding, dipteran species previously studied lack a muscle sheath covering of the secretory region of the salivary glands. Direct innervation of the salivary gland muscle sheath of tsetse would facilitate rapid deployment of saliva into the host, thus delaying a host response. Our results also suggest that the neuronal and abnormal pattern seen in viral infected glands by the *Glossina pallidipes* salivary gland hypertrophy virus (GpSGHV) is due to a compensatory increased branching of the neurons of the salivary glands, which is associated with the increased size of the salivary glands in viral infected flies. This study shows for the first time serotonin in the cell bodies of the brain and thoracico-abdominal ganglion in adult tsetse, *G. pallidipes* Austen (Diptera: Glossinidae). A hypothesis is proposed as to whether innervation of the muscle sheath covering of the secretory region of the salivary glands is present in brachyceran compared with nematoceran dipterans; and, a plea is made that more research is needed to develop a blood feeding model, similar to that in the blow flies, for elucidating the various mechanisms involved in production and deployment of saliva.

The importance of saliva production and salivation in normal feeding and its involvement in transmission of trypanosomes to the vertebrate host in tsetse flies cannot be overemphasized. Ali (1997) provided an extensive review of what is known about the aminergic and peptidergic innervation of insect salivary glands (sglds), but failed to list any references to tsetse. Studies have been reported on the techniques used to obtain tsetse saliva (Youdeowei 1975), while early reports on what is in their saliva (Patel et al. 1981) have been made. More recent studies on the tsetse sialome (Alves-Silva et al. 2010), plus composition of the saliva in both asymptomatic (Van Den Abbeele et al. 2007) and symptomatic flies by the *Glossina pallidipes* sgld hypertrophy virus (GpSGHV) (Kariithi et al. 2011) are available. Thus, it was surprising, even though suggested by the work of Alves-Silva et al. (2010), to find that no studies have been reported on whether the sglds of adult tsetse flies are innervated. Reports to date on other dipterans fail to mention whether the sglds have a muscle sheath covering the secretory region. The only other report on innervation of dipteran sglds is in the mosquito (Novak et al. 1995). In fact, most studies focus on what the mechanisms are for saliva production (Ali 1997; Baumann and Bauer 2013), what is in the saliva (Ribeiro and Francischetti 2003), but fail to mention the mechanisms for saliva deployment or delivery to the host. This study was designed to answer whether the sglds of tsetse are innervated and to provide a preliminary background for future studies on the presence of serotonin in the central nervous system (CNS). Unlike the well-studied model (Berridge and Patel 1968) for nonblood feeding flies (i.e. blowflies), there is no model system for blood feeding flies even though the sglds are extremely important and directly involved in the vectoring of various parasites and pathogens.

## Materials and Methods

### 

#### Animals

Pupae of *G. pallidipes* from a viral infected colony were received and maintained at the Insect Pest Control Laboratory of the International Atomic Energy Agency (IAEA) in Vienna, Austria. Pupae were maintained at 24°C, 70% RH, and a photoperiod of 12:12 (LD) h, as previous described in Feldmann (1994), Gooding et al. (1997), Abd-Alla et al. (2007). Both sexes were fed a sugar solution and, when designated, given heated and defibrinated bovine blood using the membrane-feeding technique of Feldmann (1994). Samples of CNS, which included the brain, cervical connective (Cc) and thoracico-abdominal ganglion (TAG) were taken for morphological observations. Moreover, specimens of both sexes, nonblood, and blood feeders (at 48 and 72 hr postfeeding), were dissected to obtain samples of normal sglds and hypertrophied sglds.

#### Light Microscopy

Samples of the CNS from normal and hypertrophied sglds were dissected in phosphate buffered saline (PBS) and immediately observed using a computerized image analysis system, which included a Zeiss light microscope (Axiophot) equipped with a video color camera (Axio Cam MRC, Arese, Milano-Italy) and imaging software (KS 300 and AxioVision).

#### Immunocytochemistry

For whole mount fluorescence immunocytochemistry of the CNS, normal and hypertrophied sglds were fixed in 4% paraformaldehyde in PBS, washed in PBST (PBS with 0.5% Triton X-100) (5 changes, 30 min each) and left in the last wash overnight at 4°C. Tissues were then blocked with 10% nonimmune goat serum/PBST (10% normal goat serum in PBST) for 1 hr with agitation before application of primary antiserum. Tissues were probed with a polyclonal anti-serotonin antiserum (Sigma-Aldrich) diluted in 10% NGS/PBST (anti-serotonin 1:1,000) for 72 hr at 4°C. Probed tissues were washed in PBST (five changes, 30 min each) and again blocked in 10% NGS/PBST for 1 hr with agitation. Tissues were soaked in fluorescein conjugated secondary antiserum (1:200) for 1 hr in darkness with agitation. Controls were run omitting the primary antibody, but photos are not provided. Finally, all samples were observed using a computerized image analysis system, which included a Zeiss light microscope (Axiophot) equipped with a video color camera (Axio Cam MRC, Arese, Milano-Italy) and imaging software (KS 300 and AxioVision).

## Results

**Fig. 1.**
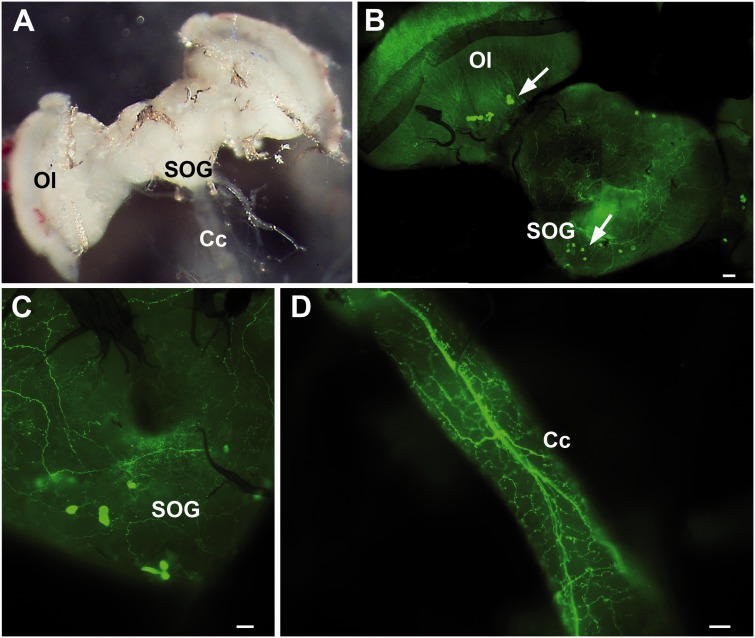
Serotonin immunoreactivity in the brain and Cc. **(A)** Brain shown for easier comparison of the serotonergic nerves presented in B and C of the SOG. **(B and C)** Cell bodies and nerve tracts are seen in the Ol area (arrow) and in SOG (arrow). **(D)** A network or neural plexus within the Cc is visible exiting the brain and going to the TAG. The presence of small boutons or varicosities being outside the brain barrier, suggests putative serotonin release sites. Bars = 10 µm

**Fig. 2.**
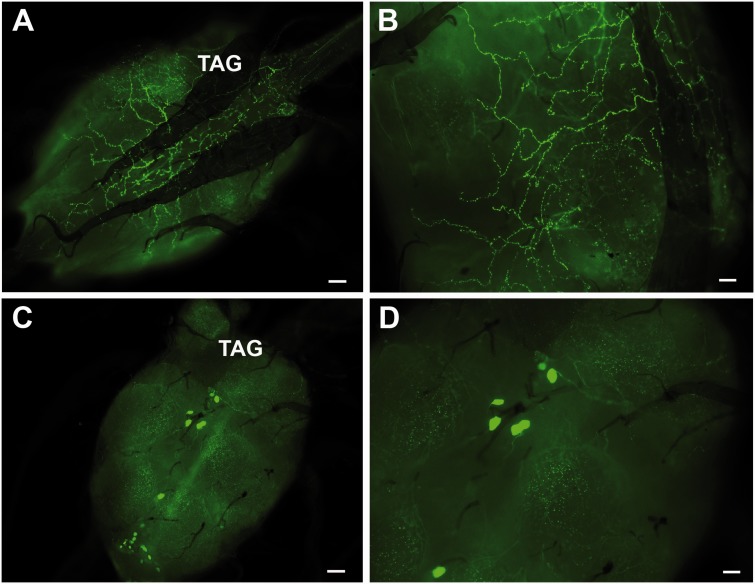
Serotonin immunoreactivity in the TAG. (**A and B)** The dorsal area of the TAG is crossed by a dense network of axons and varicosities (i.e. suggesting serotonin release sites), while in the ventral area of the TAG **(C and D)** several clusters of serotonin immunopositive cell bodies are present. Bars = 10 µm

The general layout of the CNS in *G.* *pallidipes* is described in Figure 1A. A stratified staining with serotonin immunoreactive fibers is present in all brain regions (Fig. 1B). Several immunopositive cells are observed in the protocerebrum region, in particular in the lobula (Fig. 1B). Immunoreactive cells are also present in the subesophageal ganglion (SOG), as highlighted by the high magnification in Figure 1C. A dense plexus of immunoreactive axons is evident running along the Cc (Fig. 1D) and in the dorsal surface of the TAG (Fig. 2A and B). Close examination of Figure 1D shows the plexus of the Cc with numerous varicosities, which are outside the blood brain barrier. In the ventral surface of the TAG several serotonin immunopositive cell clusters are observed (Fig. 2C and D). Controls (micrographs not shown) revealed that the immunoreactivity demonstrated in the treated specimens was serotonin.

**Fig. 3.**
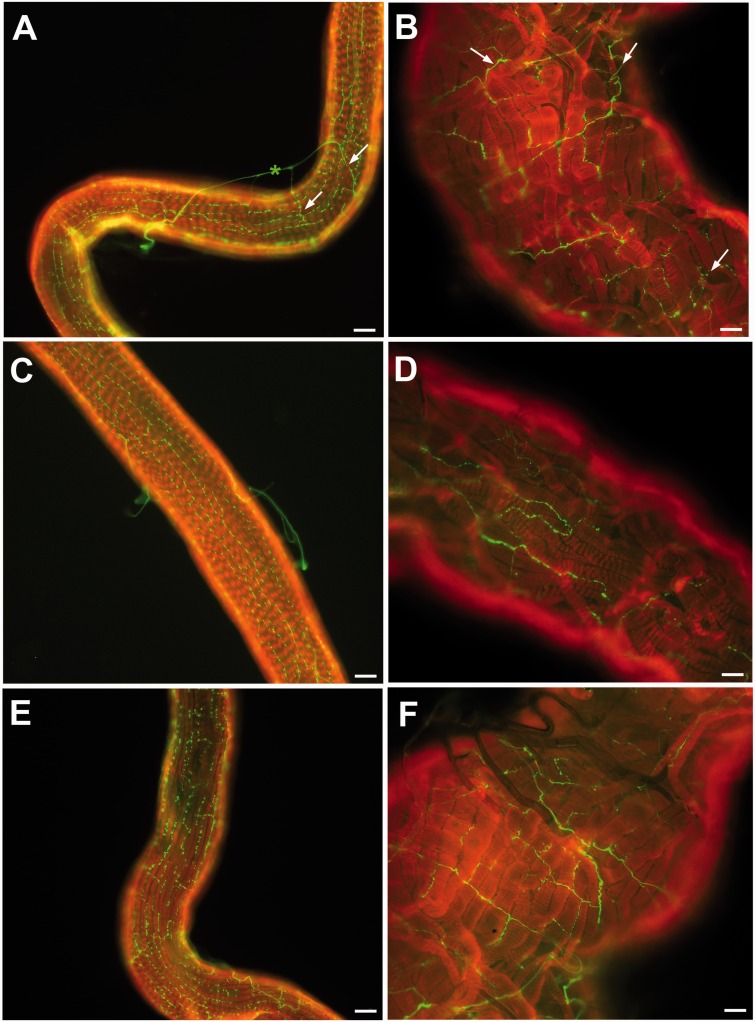
Whole mount, fluorescence immunocytochemistry of the sglds of adult female *G. pallidipes*. **(A)** Serotonin immunoreactivity is shown on the surface of the muscles of the secretory region of the sglds of asymptomatic flies prior to blood feeding. The nerve tracts generally are in parallel with the muscle fibers with some side branches (see arrows). The nerve tracts are in close association with the muscle fibers. An immunopositive axon is indicated (*) outside the muscle of the sgld. (**B)** In the hypertrophied sglds of symptomatic flies prior to blood feeding, the muscle fibers are altered (see arrows) and the nerve tracts do not follow the parallel orientation of the muscles, which is found in asymptomatic flies. **(C and D)** A similar organization and a comparable alteration of the serotonergic nerve plexus is seen in normal and hypertrophied, respectively, sglds of females 48 hr postblood meal. (**E and F)** The same pattern is seen in the sglds of asymptomatic and symptomatic flies 72 hpf. Bars = 10 µm.

Serotonin immunoreactivity is analyzed in the normal and hypertrophied sglds from female and male nonblood feeders and from flies of both sexes at 48 and 72 hr postblood feeding (hpf). Figure 3 shows the comparison between the serotonin immunolabeling in normal and hypertrophied sglds from female flies. In healthy sglds from flies prior to blood feeding, the serotonin immunoreactivity is detected throughout the secretory region of the glands and immunoreactive axons are closely associated with the muscles covering the secretory portion of the glands (Fig. 3A). Nerves run parallel to the muscle fibers with stained side branches (Fig. 3A). An external immunostained axon transmits the serotonergic signal to the nerves radiating on the surface of the sgld (see the asterisk in Fig. 3A). In the hypertrophied sglds, the muscle fibers are detached from one another and, consequently, the nerve fiber pattern is altered (Fig. 3B). Now the neural fibers don’t run parallel with the muscle fibers, but appear scattered. A similar organization and a comparable alteration of the serotonergic nerve plexus is seen in hypertrophied sglds of female flies at 48 hpf (Fig. 3C and D). Also, at 72 hpf, the serotonin innervation of normal sglds glands shows the same features as those analyzed for previous stages of flies (Fig. 3E). A similar alteration of serotonergic nerves affects hypertrophied sglds from females at 72 hpf (Fig. 3F). The serotonin immunostaining was performed also in normal and hypertrophied sglds from male flies, prior to the blood feeding and at 48–72 hpf, but the same organization of serotonin innervation and a comparable effect are observed respective to the corresponding specimens of sglds from female flies (data not shown).

## Discussion

Two groups of insects have been used as model systems for studying insect sglds (i.e. Cockroaches-Walz et al. 2006, and flies-Baumann and Bauer 2013; Röser et al. 2012). Based on these studies, we are obtaining a better understanding of the role of various amines (i.e. dopamine and serotonin for cockroaches and serotonin for the dipterans) and neural impact on saliva production. Similarities exist, as do differences between the two systems. Most studies, however, focus on secretory production and not salivation control or deployment. For these two model systems, rapid salivary delivery may not be as important as it is for blood feeding insects such as the biting flies. It is surprising when examining the literature on sglds that there is no model biting fly system; and, the overall lack of information on these potentially important vectors is so scarce. Especially since the sglds are directly involved in pathogen transmission.

Tsetse flies, like other dipterans, have tubular and not acinar sglds (i.e. cockroaches) while previous studies have shown serotonin to be the neurohormone activating saliva production (Röser et al. 2012; Baumann and Bauer 2013). In a previous report (Guerra et al. 2013), significant changes in the sglds of viral, symptomatic adults by the GpSGHV, both at the transmission electron microscope (TEM) and scanning electron microscope (SEM) level showed many changes in the glands which could have compromised normal saliva production and salivation. In the same report, no mention was made about neural control of the muscle coat covering only the secretory region of the glands. However, a later publication (Guerra et al. 2015) showed, at the TEM level, the presence of a neuron whose secretory vesicles were translucent and not opaque (i.e. suggestive of a neurotransmitter and not a neuropeptide) (Fig. 3E of Guerra et al. 2015) between two muscle fibers of the sglds. No neurons were seen in any of the cells of the secretory, reabsorptive, or proximal regions. This stimulated the present research to demonstrate neuronal stimulation of the muscle coating of the adult sglds of tsetse and to get a better idea of what the sgld hypertrophy virus has on this system.

To date, no studies have reported or demonstrated the presence of serotonin in the CNS of tsetse. Immunocytochemistry shows that clusters of serotonin immunoreactive cell bodies are present in the optic lobes (Ols), SOG, and in the ventral region of the TAG, with a neural plexus of axons running parallel to the orientation of the Cc and also in the dorsal region of the TAG of *G. pallidipes*. At one time, both *Glossina* and *Stomoxys* belonged to the Muscidae, but now *Glossina* has its own family, Glossinidae. Regardless of this change, the serotonergic patterns in the brain and TAG reported here for *G. pallidipes* are similar to those reported for *Stomoxys calcitrans* (Liu et al. 2011). Serotonergic cell bodies have been reported in a few dipteran species belonging to families other than Muscidae/Glossinidae (Nässel 1988), but no studies have been reported on adult tsetse. The presence of serotonergic fibers forming a plexus in the dorsal region of the TAG have been reported for other flies (Nässel et al. 1994; Haselton et al. 2006); it is suggested that the dorsal plexus region is a site of neurohormonal serotonin release (Nässel and Elekes 1985) and it is this hemolymph borne serotonin that is involved in stimulating the secretory region to produce saliva. It is also suggested that the TAG plexus is involved in the release of neurohormonal serotonin, which has been proposed to act on nonblood feeding dipteran sglds (Trimmer 1985; Ali 1997) and in adult *Phormia regina* Meigen to inhibit protein feeding (Haselton et al. 2009). In tsetse, the presence of the dorsal plexus suggests that sgld secretion is under the control of hormonal serotonin, like in nonblood feeding dipterans, while the muscles, presumed to be involved in dispensing luminal saliva anteriorly, are controlled by the serotonergic neural plexus (suggestion by D. R. Nässel, personal communication) as was proposed by Novak et al. (1995) for mosquitoes. Because this present study only briefly examined the presence of serotonergic neurons in CNS, one is referred to more comprehensive studies in other flies for locations, numbers, and functions for these neurons (Nässel et al. 1985).

Transmission electron microscopic evidence for the release of serotonin into the muscle area of *G. pallidipes* sglds was shown in Figure 3E of Guerra et al. (2015). This micrograph shows the terminal end of what is believed to be one of the endings of the serotonergic neurons containing translucent vesicles in the vicinity of two muscle fibers. Comprehensive TEM of all of the areas of the sglds in our previous research (Guerra et al. 2013) only showed a muscle layer surrounding the secretory region of the glands and no evidence of neural innervation of any other region was found. Our original hypothesis for the altered neural plexus of the muscles in symptomatic flies was that it resulted in the neurons breaking. In discussion with Dick Nässel (personal communication), he suggested, since the neurons appeared healthy, that what was happening was neural proliferation due to the increase in size of the sglds and not breakage (i.e. neuronal compensation for organ increase). A similar response resulting in neural proliferation has been reported for various pathological conditions and is known as neuronal proliferation (Demir et al. 2013). The article by Chen and Condron (2008) and personal correspondence with Condron, provide supporting evidence for the neuronal proliferation hypothesis. Condron noted, ‘The serotonergic arbor structure of the larval VNC (sic *Drosophila melanogaster*) grows and adds branches and varicosities as it expands from L1 to L3F (personal communication)’. Thus, this hypothesis using symptomatic flies must now be tested. Neuronal proliferation may have established new connections on the hypertrophied sgld, but the organelle disruption shown in a previous study (Guerra et al. 2013), the dissociation of the muscle fibers (Guerra et al. 2015), and the loss of collagen may produce muscle myopathy, just as it has been demonstrated in *Drosophila* mutants (Kelemen-Valkony et al. 2012). All of these abnormalities induced by the virus may affect normal saliva production and salivation.

This study is the first to demonstrate that the muscles of the secretory region of the sglds of adult tsetse flies, or any dipteran species, are innervated and by serotonergic neurons. It also confirms the suggestion of Alves-Silva et al. (2010), which provided information that suggested sgld stimulation in tsetse might involve the biogenic amine serotonin. The only other report on how the sglds are stimulated in hematophagous dipterans is the report of Novak et al. (1995) on *Aedes aegypti*. We would like to emphasize here that the studies in the Diptera have focused on control of saliva production (Baumann and Bauer 2013) and not on salivation release. The presence of muscles surrounding the secretory regions of the sgld in tsetse might be a mechanism for rapidly dispensing the saliva into the host, thus avoiding a host response. Even though muscles surrounding the sglds of mosquitoes have not been found (Ribeiro, personal correspondence) suggests that control of salivation may be different between the nematoceran and brachyceran Diptera.

Unlike in the mosquito where only the female blood feeds and the sglds are innervated (Novak et al. 1995), the sglds of both sexes of tsetse are innervated and both sexes feed on blood. Novak et al. (1995) note that, ‘5-HT-immunoreactive innervation is absent in male sglds, suggesting that 5-HT is involved in blood-feeding’. Even though we found serotonin positive neurons in both the brain and TAG, we did not follow the neural connection from the sglds to their CNS origin. The presence of numerous variscosities in the cervical connector and, being outside the blood brain barrier, suggests serotonin release sites along the connective.

In *Ae. aegypti*, Novak et al. (1995) reported that, using α-methyltryptophan (AMTP) (i.e. an established and effective serotonin depleter), females injected with AMTP and induced to salivate into mineral oil, produced less saliva and significantly lower apyrase. This led the authors to suggest that serotonin was important for salivation; and, it probably was the cause of impaired salivation. Based on their study, what is the situation in tsetse fly adults? Normal feeding in symptomatic tsetse flies infected by the SGHV has been reported as being normal for *G. pallidipes*, but abnormal for *Glossina* *morsitans centralis* (Lietze et al. 2011). Kariithi et al. (2011), however, notes that viral symptomatic *G. pallidipes* flies, 10–15 d posteclosion, have altered feeding. The difference in these reported effects on different *Glossina* species could be due to when the flies were tested (i.e. older flies being more severely impaired). In addition, further experimentation concerning the regulation of salivation by serotonin needs to be done; and, we need to know the effects of the virus on saliva output.

As Ribeiro and Francischetti (2003) reported for hematophagous dipterans to ‘steal blood’ and avoid a host response, it is essential to probe, salivate and find a vessel rapidly to successfully deploy their pharmacological salivary secretion. In order to accomplish this, we suggest some type of mechanism (i.e. for tsetse an innervated muscle coating of the sglds) should have evolved such as direct innervation for rapid ejection of saliva, as suggested here for *G. pallidipes* (i.e. serotonergic regulation). Nonhematophagous dipterans, however, have salivation regulated by serotonin, but in the dipteran species studied to date, serotonin acts as a neurohormone (Berridge and Patel 1968; Bay 1978; Trimmer 1985) and not a neurotransmitter. Mosquitoes apparently do not have muscles surrounding the sglds (Novak et al. 1995; J.M.C. Ribeiro, personal communication), which puts into question what was just said. Ribeiro (personal communication), however, suggested that the hematophagous nematoceran, versus the brachyceran Diptera, may differ as to whether the sglds are directly innervated. How the nematocerans rapidly deliver their saliva is not known. Also, it remains to be shown whether other brachyceran Diptera, such as tabanids, have muscles on the sglds and whether they are directly innervated as shown here for tsetse. Unfortunately, the extensive report by Liu et al. (2011) on *S. calcitrans*, using antibodies to serotonin, did not mention anything about innervation of the sglds.

## Concluding Remarks

This study demonstrates that the GpSGHV alters the neural plexus of the muscles surrounding the secretory region of the adult sglds of symptomatic tsetse flies. In a previous study (Guerra et al. 2015), it was shown that the disrupted muscles lose contact with one another because of what was suggested as the loss of collagen, which may result from an up-regulation of the mp-nnase gene involved in regulating interstitial collagenase production. Using the antibody to serotonin, we showed that the CNS of tsetse has serotonergic neurons and the axons from some of these probably innervate the muscle coating of the sglds. The fact that symptomatic *G. pallidipes* has altered feeding behavior suggests that a study similar to that of Lefèvre et al. (2007), which examined the effect of the trypanosome parasite on the host, should be conducted. These authors used proteomic analysis of brain tissue to compare both trypanosome infected and uninfected adults. Their results showed differences between both the levels of serotonin and also the signal transduction protein, which includes meprin (metalloendopeptidase), that could explain failure to feed in symptomatic adult tsetse, 10–15 d posteclosion (Kariithi et al. 2011), and other behaviors, in symptomatic, viral infected *G. pallidipes*. Developing a comprehensive model of both saliva production and saliva deployment (into the host or associated with nectar feeding) in hematophagous dipterans is needed.
